# Economic and quality of care evaluation of dialysis service models in remote Australia: protocol for a mixed methods study

**DOI:** 10.1186/s12913-017-2273-5

**Published:** 2017-05-03

**Authors:** Gillian Gorham, Kirsten Howard, Samantha Togni, Paul Lawton, Jaquelyne Hughes, Sandawana William Majoni, Sarah Brown, Sue Barnes, Alan Cass

**Affiliations:** 1 Menzies School of Health Research, PO Box 41096, Casuarina, NT 0811 Australia; 2 University of Sydney, Sydney, Australia; 3 Royal Darwin Hospital, Darwin, Australia; 4 Western Desert Nganampa Walytja Palyantjaku Tjutaku (WDNWPT), Alice Springs, Australia; 5 Northern Territory Department of Housing, Darwin, Australia

**Keywords:** Dialysis, Indigenous, Data linkage, Costs, Outcomes, Economic, Models of care

## Abstract

**Background:**

Australia’s Northern Territory (NT) has the country’s highest incidence and prevalence of kidney disease. Indigenous people from remote areas suffer the heaviest disease burden. Concerns regarding cost and sustainability limit the provision of dialysis treatments in remote areas and most Indigenous people requiring dialysis relocate to urban areas. However, this dislocation of people from their family, community and support networks may prove more costly when the broader health, societal and economic consequences for the individual, family and whole of government are considered.

**Methods:**

The Dialysis Models of Care Study is a large cross organisation mixed methods study. It includes a retrospective (2000–2014) longitudinal data linkage study of two NT cohorts: Renal Cohort 1- comprising approximately 2000 adults who received dialysis and Renal Cohort 2- comprising approximately 400 children of those adults. Linkage of administrative data sets from the Australian and New Zealand Dialysis and Transplant Registry, NT Departments of Health, Housing and Education by a specialist third party (SA/NT Datalink) will enable extraction of activity, financial and outcome data. Interviews with patients, clinicians and service providers, using a snowball technique, will canvass relevant issues and assist in determining the full costs and impacts of the five most used dialysis Models of Care.

**Discussion:**

The study uses a mixed methods approach to investigate the quantitative and qualitative dimensions of the full costs and outcomes associated with the choice of particular dialysis models of care for any given patient. The study includes a large data linkage component that for the first time links health, housing and education data to fully analyse and evaluate the impact on patients, their families and the broader community, resulting from the relocation of people for treatment. The study will generate a large amount of activity, financial and qualitative data that will investigate health costs less directly related to dialysis treatment, costs to government such as housing and/or education and the health, social and economic outcomes experienced by patients. This approach fills an evidence gap critical to health service planners.

**Electronic supplementary material:**

The online version of this article (doi:10.1186/s12913-017-2273-5) contains supplementary material, which is available to authorized users.

## Background

Kidney disease is a significant health issue globally and is considered by many a disease of disadvantage [1–3]. In Australia there is a steep gradient in the burden of kidney disease from urban to remote areas with people in remote areas suffering much higher levels of disease [4]. Indigenous Australians are more likely to be affected by kidney disease [5]. Nationally Indigenous Australians make up 2% of the population, although in the Northern Territory (NT) they comprise 30% of the population with the majority (70%) living in remote and very remote areas [6]. Kidney disease is particularly prevalent amongst Indigenous Territorians, who have the highest incidence and prevalence of kidney disease in Australia [7].

End stage kidney disease (ESKD) refers to the most severe stage of kidney disease when people require dialysis or a transplant to maintain life. High disease incidence coupled with improved treatment survival over the last 10 years, has led to relentless growth in the number of Indigenous Territorians with ESKD [8]. At 31st December 2014, of 654 ESKD patients, 555 (84%) were Indigenous [7].

### Evidence gap

Maintenance dialysis is disruptive for all patients and their families. However, the burden on remote area Indigenous people is particularly severe [9]. Access to dialysis treatment is limited in remote areas and the majority of patients must relocate for treatment.

Lack of health infrastructure, low access to technically skilled staff and high staff turnover in remote areas increase service provision costs [10–12]. Governments have thus been reluctant to establish staffed haemodialysis services, requiring specifically trained staff and highly technical equipment, in remote communities. Many costing studies on which these decisions are based, however, rely only on direct dialysis facility costs and do not consider the broader financial impact of dialysis provision in urban areas [13–15]. Relocation has significant consequences for the individual (loss of family support, employment, housing and costs of relocation) as well as for government services (increased demand for urban housing, social supports, transport and education). These costs can be quantified, but, to date have not been considered [16]. Therefore the true cost of this model of provision of dialysis services is likely to be underestimated.

### Impacts of relocation in Northern Territory

The majority of NT patients (75%) receive dialysis in an urban satellite facility [17]. The growing number of people requiring satellite dialysis exerts pressure on health services and urban health infrastructure. However, the large number of patients (often with families) permanently relocating for treatment is also thought to have a significant impact on other urban government services (housing, social support, education), as well as on non-dialysis-related health services.

The consequences of relocation and dislocation are pervasive [18]. Indigenous dialysis patients tend to be younger than non-Indigenous patients and still in early middle age rather than old age [7]. Typically they are active parents and community members and the loss of their leadership, cultural knowledge and skills distresses small communities [19].

In the NT, it is thought that some patients refuse dialysis rather than relocate. Others, once relocated, may prioritise family and community relationships and responsibilities over their own health needs. They might frequently miss maintenance treatments, jeopardising their health and accommodation arrangements and incurring considerable personal expense. Repeated back and forth movement between treatment location and home community has a significant financial impact on the patient, family and community [20]. Indigenous ESKD patients typically have multiple co-morbidities. A combination of poor health and dialysis requirements restricts employment options and the majority of patients receive government pensions.

Accommodation is a major issue for relocated patients and families. Public housing is in short supply with long wait lists and the higher rent of private housing is not viable for people on limited incomes. Their limited resources also make it difficult once in a house to acquire essential white-goods and furniture. Further, large numbers of visiting family can disrupt routines and create disturbances that threaten the tenancy [21]. Hostels which are furnished and provide three meals a day, offer supported urban living but leave little disposable income. More importantly, hostels do not cater for extended family and residents complain of loneliness, isolation and expense [19, 22].

With unstable accommodation, children may experience increased mobility between the urban and remote areas with extended gaps in schooling. Schools, teachers and administrators are challenged by the increased workload [23]. Importantly the impact on the long term educational outcomes and the health of this group of children is poorly understood.

Relocation for treatment places patients under extreme stress. Acceptance of treatment is influenced by the impact a treatment model has on a patient’s quality of life [24, 25]. Thus a patient’s preference for a model of care can affect treatment uptake and adherence and impact on health outcomes [26]. As the requirement for dialysis extends over many years, models of dialysis care must be sustainable, cost-effective and appropriate for the patient group and setting. Our aim is to investigate the costs and outcomes relevant to each of five selected dialysis models of care in the NT.

This paper describes the protocol for a study to evaluate the health, social and financial impact of different dialysis models of care on patients and families, health and other government services.

### Dialysis models of care (MoC) in the Northern Territory

NT Renal services are configured in a *hub and spoke* arrangement with the hubs (two tertiary hospitals) providing program oversight and specialised care. The hubs are linked to a network of dialysis facilities (*spokes*) in other urban and regional locations. The arrangement includes government as well as publicly funded non-government services. Patients are clients of both services.

The spokes comprise five principal dialysis models of care (MoC) (Table 1). Hub services are excluded. Patients move between MoC according to choice, their level of clinical stability and physical mobility and facility capacity.

**Table 1:** Principal models of dialysis care in the Northern territory

Dialysis Model	Description	Characteristics
*Model 1* Urban Satellite Unit	Larger facilities in urban areas, provides maintenance haemodialysis; training and support for self-care therapies.	All patients commence treatment here, default service when other models at capacity.
*Model 2* Regional Satellite Unit	Smaller distant facilities, often co-located with regional hospitals to access support services. Offers haemodialysis and some support for self-care patients in local community	Accepts stable, adherent patients: usually a waiting list.
*Model 3* Rural/remote Satellite Unit	Small unit distant from hub service, may co-locate with local primary health clinic to access services. Offers haemodialysis and some support for self-care therapies;	Accepts adherent, stable, physically mobile patients.
*Model 4* Community based dialysis	Community controlled services providing permanent and respite haemodialysis in small remote facilities within program of social supports.	Patient criteria less restrictive due to social supports.
*Model 5* Self-care dialysis	Multi-user facilities in remote areas for independent home haemodialysis. Single-user machines in private residences. Peritoneal dialysis carried out at home.	Patient criteria strict: adherent, clinically stable and trained to be competent and safe.

## Methods/design

The study proposes a mixed methods approach to explore the complexity of service delivery in the NT. The study includes two cohorts: Renal Cohort (RC) 1 – approximately 2000 adults who received kidney treatment in the NT between 2000 and 2014 and Renal Cohort 2 – approximately 400 children who have a parent or guardian receiving kidney treatment.

### Aims

We will link de-identified individual-level patient data from a number of sources to answer the following questions:

1. How do dialysis MoC impact upon the health, social and cultural needs of Indigenous renal patients, their families and communities?
2. What are the resource imposts of different dialysis MoC on other government and non-government organisations in both urban and remote areas?
3. What are the relative costs and outcomes of MoC that go beyond the direct costs of a dialysis treatment?

The study has four core components.

1. Creating linked administrative data sets for Renal Cohort 1 and Renal Cohort 2 across health, housing and education.
2. Analysis of service use (health, housing) and outcomes (education)
  2.1. Incidence and prevalence of ESKD by community
  2.2. Total health service utilisation by Renal Cohorts 1 and 2
  2.3. Housing demand and usage by Renal Cohort 1
  2.4. Educational outcomes of Renal Cohort 2.
3. Exploring the social, cultural and financial impacts through interviews and case studies with:
  3.1. Patients, carers and family members
  3.2. Community service providers
  3.3. Clinicians.
4. Economic analysis of total health service expenditure by:
  4.1. Dialysis MoC facility, based on financial reports
  4.2. Individuals, based on diagnosis and procedure codes.

#### Creating linked administrative data sets

**Fig. 1:**
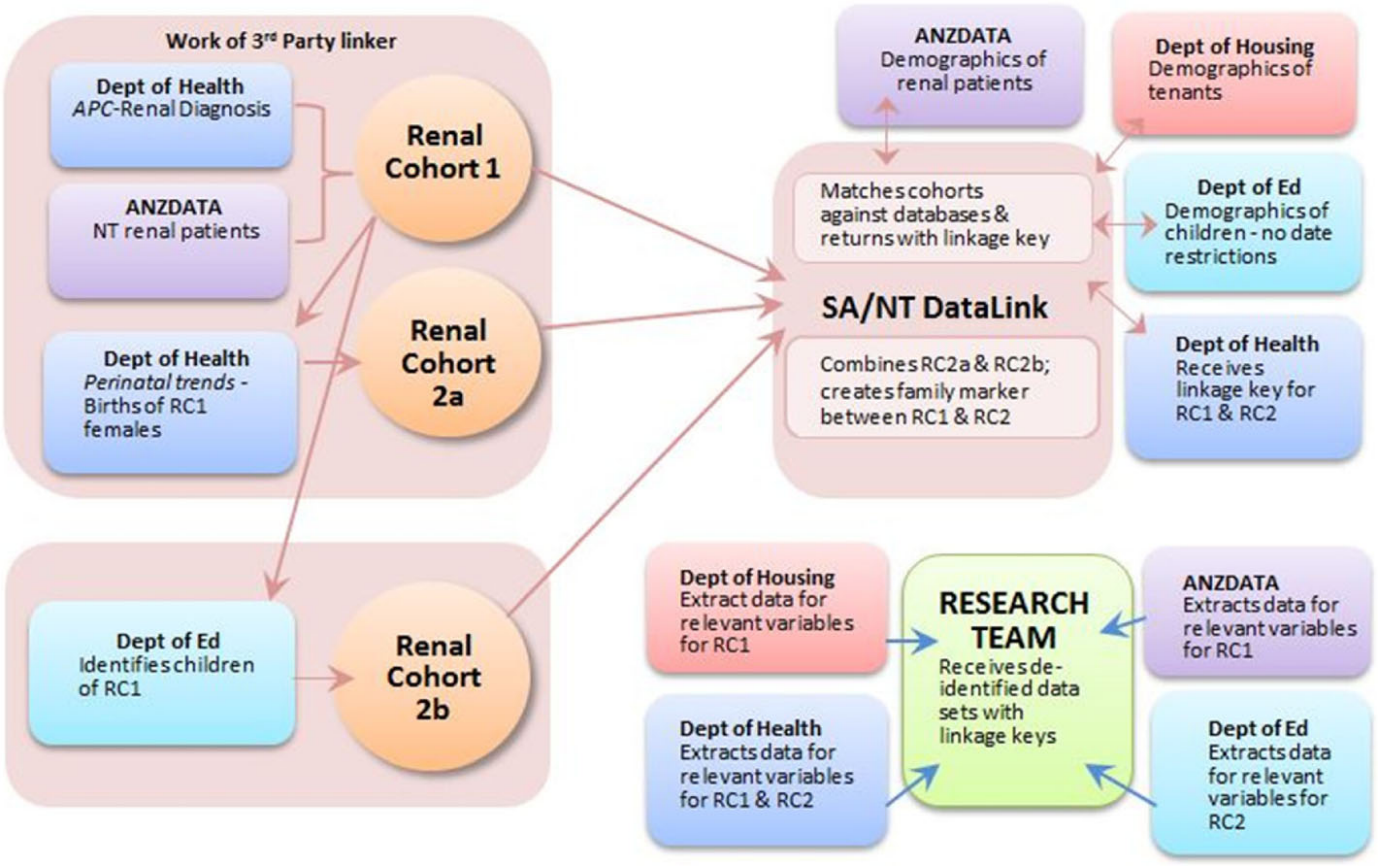
Concept map for data linkage

We will use linked administrative data sets to analyse health service usage and to determine impacts on government housing and education services. A ‘Third party linker’, a person not directly involved in the research project, will identify two cohorts using a process of deterministic linkage, followed by probabilistic linkage (Fig. 1).

##### Renal Cohort 1 (RC1)

The first cohort for the study will be identified through two data sets: the Australia and New Zealand Dialysis Transplant Registry (ANZDATA) and NT Department of Health’s (DoH) Admitted Patient Care dataset. ANZDATA is the national data repository, with records of all people receiving maintenance renal replacement treatment in Australia and New Zealand. Between 2000 and 2014, 1387 individuals were recorded in ANZDATA as having treatment in the NT.

An extraction from the DoH Admitted Patient Care data set of individuals with a diagnosis or procedure code for dialysis or transplantation (excluding acute dialysis) between 2000 and 2014 identified 1899 individuals of which 1241 were also present in ANZDATA. The additional patients are likely to comprise of individuals: a) receiving care for less than 90 days and therefore not registered in ANZDATA, b) visiting from interstate (“holiday patients”), and c) never registered in ANZDATA for other, unknown reasons. A possible total of 2045 individuals met the criteria for receiving renal replacement therapy in the NT (1387 + 1899–1241 = 2045). Coding is based on the International Classification of Diseases - 10 Australian Modification (ICD-10 AM); see Additional file 1.

##### Renal Cohort 2 (RC2)

The second cohort will be extracted from two data sets: the DoH Perinatal data set and the NT Department of Education Student Administration Management System. Children of female dialysis patients were identified through the perinatal data set. This extract (RC2a) identified 443 babies born to 217 mothers between the years 1986 and 2013.

To identify the children with a father or guardian/carer receiving treatment Renal Cohort 1 was matched against the ‘Parent/Guardian’ field in the Department of Education Student Administration Management System data set (RC2b). The two extracts, sent to SA/NT Datalink, will be combined, duplicates removed and a ‘family marker’ added to connect the child to parent/guardian. This is necessary to identify if children relocate with parents (based on the commencing community of incident patients) or remain in communities.

##### Linkage

Data linkage through SA/NT Datalink, an independent agency based at the University of South Australia will follow standard ethical systems and protocols. SA/NT Datalink has Memoranda of Understandings in place with the NT Departments of Housing, Education and Health as well as with ANZDATA for the sharing of administrative data sets for linkage purposes.

Using probabilistic matching to identify individuals common to one or more data sets, RC1 will be matched against data bases in ANZDATA and the NT Departments of Health and Housing, while RC2 will be matched against data bases in NT Departments of Health and Education. A unique identifier will link an individual across data sets. In this way de-identified information from different organisations for the same individual can be compiled.

#### Analysis of service use (health, housing) and outcomes (education)

##### Incidence and prevalence by region

To understand the demand for, and access to services we will map rates of renal treatment by locality. The ‘address’ field in the health data set, will be used to identify a patient’s usual residential community before they commenced treatment. This will be determined as the last documented address prior to the first appearance of a dialysis procedure code. This information will be mapped against regional or community population data to determine incidence and prevalence rates by region.

For each year, ‘usual community’ of commencing patients will be used to determine location and extent of demand which will then be mapped to where a patient subsequently commenced treatment. ANZDATA and hospital separation data both provide treatment location. Distance between treatment centres and ‘usual community’ will be identified and analysed. ‘Distance’ will be defined utilising the Accessibility/Remoteness Index of Australia coding [27]. In this way the accessibility of services in the NT over time will be described.

##### Tertiary health service use by Renal Cohorts 1 and 2

Relevant data sets are the Admitted Patient Care, Emergency Department and Patient Travel Management System and include activity prior to 2000. This is necessary to carry out an interrupted time series analysis of activity (before and after the intervention: that is a change in dialysis MoC). Analysis will determine whether there are patterns of associations between hospital utilisation and MoC attended.

We will examine whether the characteristics of a model (type, location, size and distance from patient’s usual community) are linked to differences in haemodialysis attendance rates and missed treatments, including unplanned hospital presentations and emergency evacuations. Uptake of activity will be determined by comparing the number of same day dialysis for each patient (ICD-10 AM code Z49.1) [28] with prescribed treatments (usually 3 treatments a week). Hospital presentations and admissions for each patient will be aggregated and allocated to the MoC the patient was undertaking. For each patient, changes in treatment modality or MoC will create discrete periods of time at risk for each MoC.

Poisson regression will be used to model the relationship of different MoC with both dialysis attendance rates and hospitalisation rates; time-to-event (survival) methods will be used to model the risk of hospitalisation and death, including Cox proportional hazard models incorporating attendance amongst other more conventional time varying co-variates and where necessary, Fine and Gray competing risks regression. Marginal structural models will be used to account for time-varying treatments (different MoC) and measured time-varying confounders. Our key measures will include: number and frequency of evacuations from communities, urgent hospital admissions, proportion of missed dialysis sessions and hospital admissions for dialysis-related complications.

Health care utilisation by Renal Cohort 2 will be examined. The ‘family marker’ and ‘usual community’ will allow analysis of whether children are relocated with dialysis patients to the urban area.

##### Housing demand and usage by Renal Cohort 1

Linking Renal Cohort 1 to housing datasets will provide a better understanding of the demand for, and usage of, housing stock by dialysis patients. Analysis will identify the extent of waitlists and wait times, demand by relocated patients, type of accommodation requested and allocated, number of house residents, location of residence, duration of tenancy, mobility of tenants and use of interpreters.

The analysis will be supplemented with information from interviews with patients, hostel managers, Indigenous housing organisations, community service providers and social support staff.

##### Educational outcomes of Renal Cohort 2

We will examine school attendance rates and educational outcomes of children who have a parent on dialysis to determine if there are differences for those that accompany relocated patients. Data sources from the NT Department of Education that are relevant for this project include the National Assessment Program Literacy and Numeracy scores, Kids in Town Engaged in School program and Language Background Other than English data.

#### Exploring social, cultural and financial impacts through interviews and case studies

The approach to the qualitative inquiry is guided by principles for conducting research with Indigenous peoples – reciprocity, respect, equality, survival and protection, and spirit and integrity [29]. Relationship building will be a priority. We will work through existing structures and processes to engage Indigenous people in the research process to strengthen these structures and minimise the burden on Indigenous people.

We will use an ethnographic approach to explore participants’ views and understand the impact of the different MoC on people including people on dialysis, their families and communities as well as a range of health professionals and service providers.

Indigenous researchers (patients and carers) will be employed to assist with community brokerage and contribute to the qualitative data collection, analysis and interpretation. The study will focus on developing the research capacity of the Indigenous researchers.

*Interviews:* We will conduct in-depth interviews with (i) dialysis patients their family and community members; (ii) health professionals and other agencies/organisations with specific relationships to kidney and community-based patients; and (iii) social and other service providers. Interviews will be semi-structured and interviews with patients and family members will take a narrative approach to encourage participants to share their story of being on dialysis. The interviews will explore factors that influence patient/family treatment choice; accessibility and acceptability of services; whether models meet patient needs; suggested improvements; perceived social, cultural and financial impacts of treatment models on patient, family and community, particularly in relation to relocation for dialysis treatment. Interpreters will be used whenever necessary.

We will use a key informants and snowball sampling strategy to identify potential participants for interview [30]. All interviews will be recorded (with permission), transcribed verbatim and NVivo software will be used to organise and code data.

In addition, workshops with senior Indigenous people will be facilitated to explore the lived experience of dialysis and its social, cultural and financial impacts. These workshops will also explore the concept of quality of life and what makes a good life on dialysis from an Indigenous perspective.

*Case studies:* Case studies will be developed to illustrate and contextualise key issues relating to the social, cultural and financial impacts of the different MoC.

An inductive analysis of the qualitative data will identify key themes relating to the provision of the different MoC which will not only contribute to answering the research questions and emerging findings during the study but will inform the analysis and interpretation of the quantitative data.

#### Economic analysis of total health services expenditure

We will develop a zero-based cost template to ensure comprehensive cost data capture and collection. Direct financial expenditure (2008–2014) from DoH reports and non-government organisation will be analysed split by dialysis facility and MoC. Data will be reported in a single base year (i.e. 2014 dollars) and adjusted for each preceding year.

Financial data will include:

1. Direct costs attributed to the service delivery of each model;
2. Indirect costs such as support services provided by the hub or hospital;
3. Capital establishment costs including budget over-runs where relevant; and
4. Other cost considerations include staff accommodation infrastructure, contingency planning requirements (backup generator, cyclone evacuation).

Initially costs will be allocated to each model for each location.

Mapping patient attendance against the operating costs for each service over the corresponding periods, will give a series of price per treatments, per year, per dialysis MoC.

Secondly, costs will be attributed to each activity identified using the National Efficient Price cost weights [31]. The National Efficient Price cost-weight includes adjustments for remoteness and Indigeneity but does not include costs funded from other Australian Government programs such as the Pharmaceutical Benefit Scheme. In the NT, maintenance dialysis patients’ medications are covered by the DoH in recognition of their low disposable income. Pharmacy expenditure is linked by medical record numbers to individual patients as are travel costs (e.g. to or from communities or interstate for treatment).

We will attribute all hospitalisation, pharmacy and travel expenditure incurred by individual patients to their respective model of care at each point in time. Our interrogation of the data will allow us to calculate total utilisation costs for each patient from dialysis commencement to 2014.

In turn, we can then create a cost model for each MoC including identifying critical gaps in expenditure capture that may impact on the robustness of the cost study. Using this approach, we can also compare and contrast specific MoC costs from our study with existing cost studies based only on direct dialysis or facility costs.

## Discussion

The study uses a mixed methods approach to investigate the quantitative and qualitative dimensions of the full costs and outcomes associated with particular dialysis models of care for the largely remote living Indigenous patients of the NT. Patient and staff commentary coupled with detailed data analysis will support a more accurate identification and quantification of the full range of dialysis impacts. These impacts include health costs less directly related to dialysis treatment, costs to government such as housing and/or education and the health, social and economic outcomes experienced by patients. This approach fills an evidence gap critical to health service planners. Findings will be dependent on the quality and completeness of data as well as the timeliness of data delivery.

## Additional file

Additional file 1: International Classification of Diseases -10 AM Codes related to renal replacement therapy used for identification of Renal Cohort 1. Table of ICD codes and descriptors (DOCX 14 kb)

## Abbreviations

ANZDATA: Australian & New Zealand Dialysis and Transplant Registry
DoH: Department of Health (NT)
ESKD: End Stage Kidney Disease
HREC: Human Research Ethics Committee
ICD-10 AM: International Classification of Diseases – 10 Australian Modification
MoC: Models of Care
NT: Northern Territory
RC: Renal Cohort
SA/NT Data Link: South Australia & Northern Territory Data Link
