# Supplementary material for: Economic and quality of care evaluation of dialysis service models in remote Australia: protocol for a mixed methods study
Source: BMC Health Serv Res. 2017 May 3;17:320. doi: 10.1186/s12913-017-2273-5 (PMC5415781; doi:10.1186/s12913-017-2273-5)
Supplement: Additional file 1: — International Classification of Diseases -10 AM Codes related to renal replacement therapy used for identification of Renal Cohort 1. Table of ICD codes and descriptors (DOCX 14 kb) [file 12913_2017_2273_MOESM1_ESM.docx]

Additional File 1 : International Classification of Diseases-10AM Codes related to renal replacement therapy used for identification of Renal Cohort 1

| DRG | AO9A | Renal transplant plus pancreas |
| --- | --- | --- |
|  | AO9B | Renal transplant without pancreas |
|  | L61Z | Same day dialysis |
|  | Z49.0 | Preparatory care without treatment - adjustment/fitting of catheter |
|  | Z49.1 | As above |
|  | Z49.2 | Peritoneal |
|  | T85.71 | Complication peritoneal dialysis infection or inflammation |
|  | T86.1 | Kidney transplant failure rejection |
|  | Z94.0 | Kidney transplant status |
| Proc | 13100-00 | Haemodialysis |
|  | 13100-03 | Intermittent haemodiafiltration |
|  | 13100-06 | Peritoneal dialysis - short term |
|  | 13100-07 | Intermittent peritoneal dialysis long term |
|  | 13100-08 | Continuous peritoneal dialysis long term |
|  | 90352-00 | education for home dialysis |
